# Perception of pharmacological equivalence of generics or biosimilars in healthcare professionals in Vienna

**DOI:** 10.1007/s00228-023-03603-3

**Published:** 2023-12-22

**Authors:** Lukas Binder, Markus Zeitlinger

**Affiliations:** 1 Medical University Vienna, Vienna, Austria; 2 Department of Clinical Pharmacology, Medical University Vienna, Vienna, Austria

**Keywords:** Generic drugs, Biosimilars, Vienna, Physicians, Nurses, Perception, Knowledge

## Abstract

**Purpose:**

Due to constantly rising therapy costs, biosimilars and generic drugs have gained tremendous importance through recent decades. Nevertheless, the acceptance among healthcare workers regarding biosimilars and generic drugs in previously published international studies is considerably lower than the scientific data on equivalent safety and efficacy would suggest. The aim of this questionnaire-based survey was to determine the perception and knowledge regarding generic drugs and biosimilars by medical professionals from different healthcare facilities in Vienna, Austria.

**Methods:**

The online questionnaire was sent to public and religious hospitals in Vienna, including the university hospital “Vienna General Hospital.” In addition, doctors’ offices were reached by sending out the questionnaire in the weekly news of the Vienna Medical Association.

**Results:**

A total of 282 physicians and 311 graduated nurses took part in the study. 63% and 62% of the participants were convinced that generic respective biosimilar drugs were clinically equivalent to the original reference drug. On average, 1.6 out of 4 knowledge questions were answered correctly about generics, while only 0.87 out of 4 questions were answered accurately about biosimilars.

**Conclusion:**

The results of this study support the outcome from previous surveys demonstrating that a large proportion of healthcare professionals is still skeptical about generics and biosimilars. According to the results of this study, better education of the medical staff might ensure greater acceptance of these types of drugs.

**Supplementary Information:**

The online version contains supplementary material available at 10.1007/s00228-023-03603-3.

## Introduction

After the patent for an originally developed drug has expired, unbranded versions such as generic drugs and biosimilars can enter the market. The duration of drug patents is uniformly regulated worldwide lasting 20 years from the date of patent application. While generics and biosimilars derive from fundamentally different drugs, they serve the same purpose. Due to their significantly lower costs compared to the branded drugs, they are able to reduce the financial burden on the health system and promote medical innovation and research into new medicines by ensuring more competition on the pharmacological market [1, 2].

In Austria, doctors in their own practice can specifically prescribe biosimilars or generics. The dispensing of medicines in the inpatient sector in hospitals is regulated by the respective umbrella organization, but prescriptions for home medication are also issued individually by the doctors. Substitution, i.e., the automatic exchange of one medication for another therapeutically equivalent drug by the pharmacist, is not permitted in Austria [3].

Generic drugs are derived from chemically manufactured medicines. They contain the same active ingredient in an identical dosage and a comparable dosage form. However, excipients, packaging, shelf life, and the manufacturing process can differ from the original product. The manufacture processes of generic drugs is subject to the same regulations as those for original drugs [4, 5].

The brand name counterpart of biosimilars are biological medicines, often referred to as biopharmaceuticals or biologics. In contrast to chemical medicines, biologics are produced by a living organism. Therefore, a biologic is made of large, complex proteins, whereas the generic consists of small, rather easy reproducible molecules. Because of the size and complexity, it is not possible to produce biopharmaceuticals chemically [6, 7]. The manufacturing process of biosimilars is therefore much more complex and expensive than that of generics.

Generic drugs as well as biosimilars must be tested for pharmacological bioequivalence compared to the reference originator drug in order to get approval to enter the market within the European Union [8, 9]. The vast majority of clinical data from randomized double-blind studies also confirms no significant differences of clinical outcomes in patients compared to the original drugs.

However, previous studies have shown that healthcare professionals around the world still express various concerns about generics and biosimilars and are sometimes reluctant to prescribe those medicines. Frequently addressed concerns are related to quality, safety, or efficacy of generics and biosimilars in general, while more specific worries refer to switching patients from therapy with the brand name drug to the respective generic or biosimilar drug [10, 11].

Although clinical evidence is continuously growing and prescription numbers of generics and biosimilars are rising, recent studies do not indicate that the reputation of generics and biosimilars among physicians is increasing [10, 12–15]. Furthermore, most studies are targeting physicians only and other important health care workers like nurses are often left out. For the first time in Vienna, Austria, this study is intended to provide an overview of the opinions and knowledge of doctors and nurses regarding generics and biosimilars. Additionally, the collected data will be compared with international results from other studies.

## Methods

### Study design

The concept of the study is a questionnaire-based, anonymous exploration of knowledge and acceptance of healthcare workers regarding generics and biosimilars. The questionnaire was created using the online provider “SoSci Survey,” a professional online platform for creating and evaluating questionnaires. It is developed by universities, used for research purposes, and uses strict data protection criteria. The questionnaire was sent via link to hospitals, healthcare facilities, and doctors’ offices in Vienna. It was accessible online for a total period of almost 3 months, from 07.04.2021 to 01.07.2021.

### Participants

Participants consisted of physicians as well as nursing staff coming from all recognized disciplines of the Austrian medical training regulations. All participants must have finished their respective medical education—physicians must have obtained the diploma of medicine; nursing staff must be qualified. Participants working in healthcare facilities outside of Vienna have been excluded from the analysis.

### The questionnaire

The questionnaire was written in German and featured 30 questions in the format of multiple choice or single choice regarding demographic characteristics, attitudes, and perceptions as well as knowledge about generics/biosimilars. The main part was thematically divided into “generics” and “biosimilars.” The participants were asked about their opinion regarding the therapeutic equivalence of the brand name drugs to their respective generics or biosimilars as well as their general prescribing habits. After a self-assessment of background knowledge on this topic, they then must answer four clinically relevant knowledge questions each relating to generics and biosimilars. Finally, participants could declare whether more advanced training on this topic is desired. An English version of all questions is enclosed as a supplement in tabular form (Table S1).

### Statistical analysis

For the statistical analysis, four groups were defined among the surveyed participants:

- Physicians from the university hospital “Vienna General Hospital”
- Physicians from other hospitals in Vienna
- Physicians from doctors’ offices in Vienna
- Qualified nursing staff from all listed health care institutions in Vienna

The aim of the statistical analysis was to compare the results of the survey within those four groups. It was to determine whether significant differences in the responses exist between the four groups related to the opinion on the equivalence of original drugs and generics/biosimilars. The participants could either agree with the clinical equivalence with “yes” or deny it with “no.” Additionally, the answers on generics and on biosimilars must be considered separately. Each of these questions was tested for pairwise differences using a chi-square test between two of each of the four groups. With four groups surveyed, this resulted in six pairwise comparisons per question.

The secondary objective of the analysis was to determine whether there are any differences in the four groups regarding knowledge about generics or biosimilars. To each of the two topics, the participants got to answer four single choice questions to test their basic level of knowledge. To prevent participants from guessing, each question provided the option of checking “don’t know.” The answers then resulted in a knowledge score—one for generics and one for biosimilars—derived from the number of correctly answered questions—ranging from 0 to 4. The scores were then tested for significant differences within the four groups. Since this involved nominal and ordinal variables, pairwise Mann–Whitney *U* tests were applied. With four groups examined, this resulted in six pairwise Mann–Whitney *U* tests.

Further statistical analyses focused on the examination of potential correlations between answers of the participants. For this purpose, correlation analyses were performed using the coefficient Kendall tau-c.

In all cases that were subject to multiple testing, a correction of the alpha value has been performed using the Bonferroni method.

Statistical analysis of the data was performed using the programs SPSS Statistics 28 (IBM, Armonk, NY, USA) and Microsoft Excel.

#### Declaration of the partially adjusted study population in the sector of biosimilars

The first question regarding biosimilars is a filter question and explores whether participants know the term biosimilars at all. Those participants who chose the answer “No, I don’t know this group, nor can I imagine anything about this term” would skip all further questions on the topic of biosimilars.

For this reason, the results of the survey on the topic of biosimilars partially do not refer to the entire study population *n*, but only to the population that was either slightly or completely familiar with biosimilars = *x*, since no adequate statements could be expected from the participants, who did not know the group of biosimilars at all. Thus, an adjusted study population of *x* instead of *n* is considered here; the percentages refer to *x*.

However, all results of the knowledge test as well as self-assessment of knowledge still refer to the basic population, since the mentioned respondents, who declared to not know biosimilars at all, cannot have any knowledge about biosimilars. Thus, these participants are assigned a self-assessment of knowledge of 0 points as well as a knowledge score of 0.

## Results

### Demographic overview

In total, the questionnaire was accessed 1711 times. 759 surveys were started, of which 607 questionnaires were validly completed. Due to the previously defined exclusion criteria, 11 questionnaires were excluded from the study. Consequently, this leads to a valid population of 596 participants, consisting of 282 (47.3%) physicians and 314 (52.7%) participants of the nursing staff.

Divided into the previously defined 4 study groups, this results in:

- 67 physicians from the university hospital “Vienna General Hospital”
- 158 physicians from other hospitals in Vienna
- 57 physicians from doctors’ offices
- 314 qualified nurses

A detailed demographic breakdown is presented in supplement Table S2.

Survey times have been recorded and then reviewed using the “Relative Speed Index (RSI).” This indicates how much faster an individual questionnaire was completed than the median of all other completed questionnaires in the collective. If the RSI value is above 2.0, the specific questionnaire should be analyzed more critically, and the respective times of the individual pages should be examined. The median time for finishing the whole questionnaire was 5 min and 58 s, while the average time being 6 min and 23 s. None of the questionnaires had to be excluded due to an abnormal speed index.

### Results generics

Overall, 70.2% of physicians and 56.7% of nurses (63.1% of the total study population) endorsed the equivalence of generic drugs and their originator drugs (Table 1). The chi-square test revealed significant differences in responses between hospital physicians and certified nurses (*p* = 0.004). No significant differences were found among the other groups tested.

**Table 1:** Cross-tabulation Study group * Generic equivalence. Listing of the participants’ votes on the equivalence between generic and original products subdivided into the previously defined study groups

			Equivalence		Total
			Yes	No	
Study group	University hospital	Number	48	19	67
		% of university hospital	71.6%	28.4%	100.0%
	University hospital	Number	111	47	158
		% of other hospitals	70.3%	29.7%	100.0%
	Doctors’ practices	Number	39	18	57
		% of doctors’ practices	68.4%	31.6%	100.0%
	Nursing staff	Number	178	136	314
		% of nursing staff	56.7%	43.3%	100.0%
Total		Number	376	220	596
		% of all working areas	63.1%	36.9%	100.0%

On a scale of 1–10, the participants rated their own level of knowledge regarding generic drugs at an average of 6.8 points. On average, the group of physicians rated themselves higher ($\overline{x }$ = 7.4) than the group of graduate nurses ($\overline{x }$ = 6.3).

Out of the four knowledge questions asked about generic drugs, an average of 1.6 questions were answered correctly (Table 2). Physicians performed better on average than the nursing staff ($\overline{x }$ = 1.97 versus 1.26). Between the four defined study groups, medical staff from the university hospital achieved the best results: $\overline{x }$ of the achieved knowledge score by physicians from the university hospital = 2.16; doctors’ offices = 2.02; other hospitals = 1.87; nursing staff = 1.26.

**Table 2:** Cross-tabulation Study group * Generic knowledge score. Overview of correctly answered knowledge questions about generics divided into the previously defined study groups

			Generic knowledge score					Total
			0	1	2	3	4	
Study group	University hospital	Number	3	17	20	20	7	67
		% of university hospital	4.5%	25.4%	29.9%	29.9%	10.4%	100.0%
	Other hospitals	Number	19	38	51	44	6	158
		% of other hospitals	12.0%	24.1%	32.3%	27.8%	3.8%	100.0%
	Doctors’ practices	Number	3	19	17	10	8	57
		% of doctors’ practices	5.3%	33.3%	29.8%	17.5%	14.0%	100.0%
	Nursing staff	Number	72	124	85	29	4	314
		% of nursing staff	22.9%	39.5%	27.1%	9.2%	1.3%	100.0%
Total		Number	97	198	173	103	25	596
		% of all working areas	16.3%	33.2%	29.0%	17.3%	4.2%	100.0%

Using Mann–Whitney *U* test procedure, significant differences in knowledge scores were found between:

- Physicians from the university hospital and nurses (*p* < 0.001, *r* = 0.309)
- Hospital physicians and nurses (*p* < 0.001, *r* = 0.274)
- Practice-based physicians and nurses (*p* < 0.001, *r* = 0.234)

Between the individual groups of physicians, testing revealed no significant differences in knowledge about generic drugs.

76.2% of physicians were familiar with the differences in composition between generics and brand name drugs (nurses: 63.7%); 52.1% knew that stricter guidelines apply to the approval of generics with a narrow therapeutic range (nurses: 29.6%). The approval procedure could be correctly named by 39% (nurses: 13.4%). The greatest uncertainty existed in the correct identification of the applicable pricing model of generics: 29.8% of the physicians were familiar with the established tiering scheme (nurses: 19.7%). Table S3 provides a detailed overview of all four questions regarding generics and the distribution of all answers selected.

69.1% of all physicians surveyed would not have preferred the original drug for themselves in the event of an illness, while 24.8% clearly would have preferred the original over the generic. There is a distinct difference when compared with the attitude of the nursing staff: 51.9% of nurses would clearly prefer to take the original rather than the generic.

**Fig. 1:**
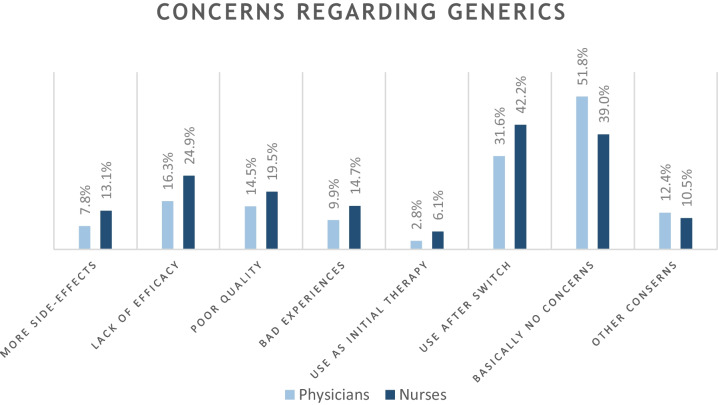
Expressed concerns about generics divided into physicians and qualified nursing staff. Percentages refer to the totality of the respective study population of physicians and nursing staff

Forty-five percent of respondents had no fundamental concerns when using generics in the clinic. The greatest doubts about generics were expressed if a patient’s therapy was switched from an original drug to the generic (37.1%); if generics were established as initial therapy, far fewer participants expressed concerns (4.5%). Regarding the general characteristics of generics, 20.8% feared lower efficacy, 17.1% poor quality, and 10.6% more side effects. 12.4% have had poor experiences with generics throughout their professional careers. Other concerns about generics mainly related to worse patient compliance due to frequent preparation changes (3.5%) and an increased risk of application and prescribing errors (2.1%). The breakdown of the concerns expressed by physicians and nurses can be seen in Fig. 1.

### Results biosimilars

14.5% of the physicians and 57.0% of the qualified nursing staff had never heard of biosimilars and were unable to associate anything related to this term, despite listing some of the most commonly used representatives within Austria below the question. 39.7% of physicians and 26.4% of nurses had at least heard of or have had some brief contact with biosimilars at some point in their careers. Ultimately, 45.7% of physicians and 16.6% of nurses declared that they were actually familiar with biosimilars.

Subsequently, the analysis population is adjusted. Participants who had never heard of biosimilars were not asked any questions about their attitude toward these drugs, as no qualified statements are possible in this case. 220 participants had never heard of biosimilars, so the adjusted population is 596 − 220 = 376. However, all results of the knowledge check and self-assessment of knowledge still refer to the original population.

64.7% of the physicians surveyed were convinced of the equivalence of biosimilars compared to their originator products; out of the graduate nursing staff, the figure was 58.5%. This results in an overall affirmation of equivalence by 62.5% of all respondents (Table 3). There is no significant difference between the 4 study groups.

**Table 3:** Cross-tabulation Study group * Biosimilar equivalence. Listing of the participants’ votes on the equivalence between biosimilar and original products subdivided into the previously defined study groups

			Equivalence		Total
			Yes	No	
Study group	University hospital	Number	36	24	60
		% of university hospital	60.0%	40.0%	100.0%
	Other hospitals	Number	87	46	133
		% of other hospitals	65.4%	34.6%	100.0%
	Doctors’ practices	Number	33	15	48
		% of doctors’ practices	68.8%	31.3%	100.0%
	Nursing staff	Number	79	56	135
		% of nursing staff	58.5%	41.5%	100.0%
Total		Number	235	141	376
		% of all working areas	62.5%	37.5%	100.0%

Again, physicians rated their personal knowledge higher than the nursing staff. Regarding biosimilars, physicians assess their overall knowledge at an average of 3.6 on a scale of 1–10; the average for nursing staff was 2.1 (average of all participants: 2.8).

The actual average knowledge score achieved out of 4 questions about biosimilars was 0.87 (Table 4). Again, physicians from university hospitals, proportionately, were able to answer most questions correctly ($\overline{x }$ = 1.58), followed by physicians from doctors’ offices ($\overline{x }$ = 1.31), physicians from other hospitals ($\overline{x }$ = 1.27), and nursing staff ($\overline{x }$ = 0.44). All 4 groups were once again tested among themselves for differences in knowledge score; significant differences were found between the following groups:

- University hospital physicians and nursing staff (*p* < 0.001, *r* = 0.400)
- Hospital physicians and nursing staff (*p* < 0.001, *r* = 0.353)
- Practice based physicians and nursing staff (*p* < 0.001, *r* = 0.322)

**Table 4:** Cross-tabulation Study group * Biosimilars knowledge score. Overview of correctly answered knowledge questions about biosimilars divided into the previously defined study groups

			Biosimilars knowledge score					Total
			0	1	2	3	4	
Study group	University hospital	Number	18	16	15	12	6	67
		% of university hospital	26.9%	23.9%	22.4%	17.9%	9.0%	100.0%
	Other hospitals	Number	62	34	29	23	10	158
		% of other hospitals	39.2%	21.5%	18.4%	14.6%	6.3%	100.0%
	Doctor’s practices	Number	21	14	10	10	2	57
		% of doctors’ practices	36.8%	24.6%	17.5%	17.5%	3.5%	100.0%
	Nursing staff	Number	228	44	32	9	1	314
		% of nursing staff	72.6%	14.0%	10.2%	2.9%	0.3%	100.0%
Total		Number	329	108	86	54	19	596
		% of all working areas	55.2%	18.1%	14.4%	9.1%	3.2%	100.0%

“Biosimilars are derivative products of biopharmaceuticals, however not with structurally identical, but highly similar active ingredient”—this definition of biosimilars could be correctly assigned by 50.7% of the physicians (nurses: 25.8%). The manufacturing process of biosimilars as well as the structural difference to generics could be correctly selected by 32.3% of the physicians (nurses: 7.6% and 5.1%, respectively). 19.1% were aware that the costs associated with the manufacturing process of biosimilars are higher than those of generics (nurses: 5.7%)—Table S4.

37.2% of respondents would prefer to use the original product themselves when ill. 37.5% had no preference between original and biosimilar. 25.3% did not want to make any statement in this regard. In more detail and divided between physicians and nurses, the majority of physicians would not favor any product, while the majority of the nursing staff would prefer to take the original product (physicians: prefer original yes = 29.5%, prefer original no = 44.4%; nurses: prefer original yes = 51.1%, prefer original no = 25.2%).

**Fig. 2:**
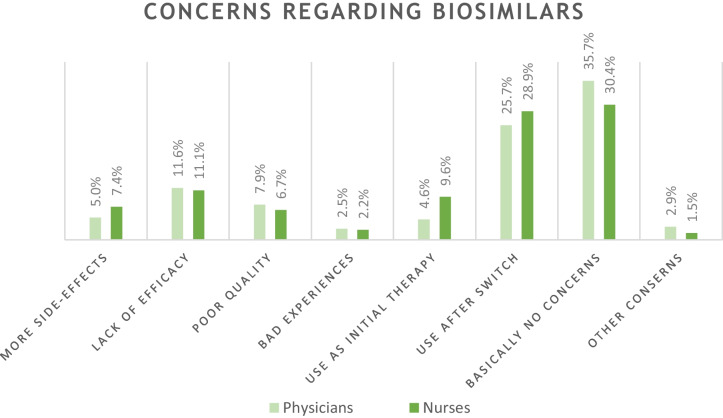
Expressed concerns about biosimilars divided into physicians and qualified nursing staff. Percentages refer to the totality of the respective subgroups of physicians and nursing staff

Compared to generics, considerably fewer participants seem to have no concerns about biosimilars (33.8%). 26.9% had concerns about switching biosimilars; 6.4% expressed doubts about using them as initial therapy. Lower efficacy was feared by 11.4% of respondents, poor quality by 7.4%, and more side effects by 5.9%. Bad experiences with biosimilars in their patients had been experienced by 2.4% of the healthcare professionals. Compared to concerns about generics, a significantly larger proportion of participants (29.0%) selected the “don’t know” option (Fig. 2).

### Further results

Regarding the prescription of generics, 21% of physicians stated that they prescribe generics as often as possible. Fifty-eight percent, thus the majority of all physicians, usually establish generics as initial therapy for their patients, but are hesitant to switch an existing therapy. 14.6% described themselves as “reluctant” and prescribe generics only after detailed individual consideration and indication. Five percent of physicians try to avoid generics.

**Fig. 3:**
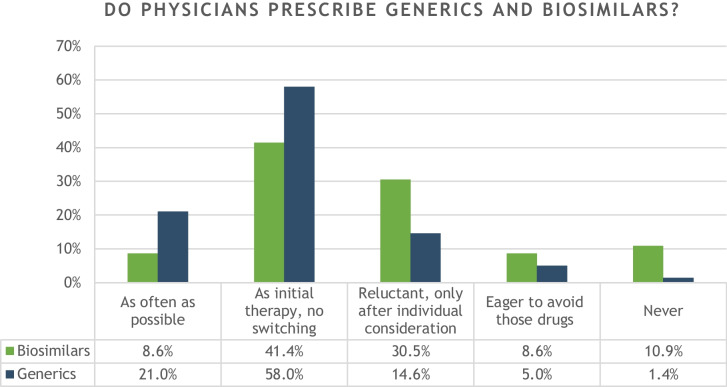
Overview of physicians’ statements on their willingness to prescribe generics and biosimilars. The relative percentages refer to the respective population of physicians surveyed (generics *n* = 281; biosimilars *n* = 241)

In the case of biosimilars, physicians were generally more critical: of the population with sufficient prescribing practice, a significantly larger proportion was reluctant to prescribe biosimilars (30.5%); likewise, comparatively more physicians were committed to mostly avoiding biosimilars (8.6%) or never using them (10.9%). 8.6% of physicians prescribe biosimilars as often as they can. This information is visualized in Fig. 3.

**Fig. 4:**
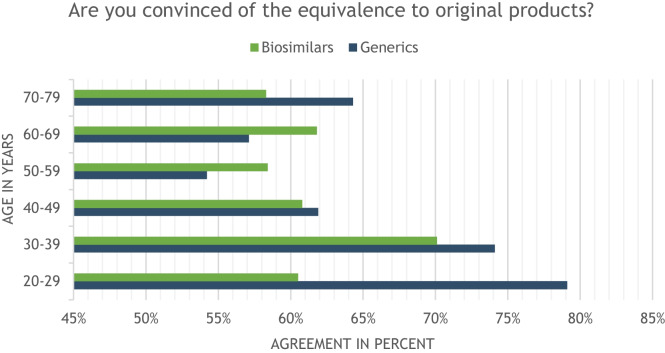
Agreement on the equivalence of generics/biosimilars in relation to the respective originator products, separated by age groups of participants

Older participants appear to be slightly more skeptical of generics than younger participants. This is shown by the correlation analysis between increasing age and endorsement of 1:1 interchangeability between generic and original drugs on a scale of 1–11 (Kendall tau-c = −0.111, *p* < 0.001). Whereas 76% in the 20–40 year age group endorsed generic equivalence, only 55% did so among 50 + participants. Regarding biosimilars, the differences were not as pronounced, with 67% of 20–40 year-olds and 59% of participants age 50 + agreeing with the equivalence of biosimilars and originator drugs (Fig. 4).

The knowledge scores of the individual age groups do not differ significantly from one another in either subject area.

Further statistical analyses indicate that opinions and knowledge differ between the groups of specialists. On average, surgeons achieved by far the lowest knowledge score for both generics and biosimilars compared with other specialty groups. Internists, on the other hand, achieved the highest scores in both categories (Table 5).

**Table 5:** Overview of correctly answered questions about generics/biosimilars separated by medical specialty

Medical specialty	Generics knowledge score		Biosimilars knowledge score	
	Mean	*n*	Mean	*n*
Internal medicine	2.26	74	1.62	74
Surgery	1.43	21	0.76	21
General medicine	2.19	42	1.36	42
Other medical specialties	1.82	128	1.26	128
Total	1.97	282	1.34	282

Surgeons also stood out when it comes to the question of equivalence. Overall, 70.2% of physicians were convinced of the equivalence of generics—among surgeons, the figure was only 47.6%. The two specialty groups with the highest knowledge scores on generics were also the groups with the proportionally highest conviction of equivalence (internal medicine = 73.0%; general medicine = 76.2%).

Likewise, in the field of biosimilars, the two specialty groups with the least knowledge—surgery and other specialties—were the groups that proportionally agreed at least with the equivalence of biosimilars (57.1% and 54.6%, respectively).

Knowledge seems to have an impact on opinions about generics and biosimilars. The response to the question “Are generics/biosimilars 1:1 interchangeable with the respective original product?” on a scale from 1 (not at all) to 11 (fully) is a parameter for further exploration of the conviction of equivalence. Statistical testing of the entire study population using Kendall tau-c shows significant correlations between the knowledge score and the conviction of clinically safe interchangeability of generics (Kendall tau-c correlation: 579 cases, *r* = 0.151, *p* < 0.001).

In the case of biosimilars, significance was found, but the correlation coefficient and therefore also the effect size is less than 0.1. So statistically, despite the significant result, there is in fact no decisive correlation between the variables (Kendall tau-c correlation: 298 cases, *r* = 0.086, *p* = 0.045).

**Fig. 5:**
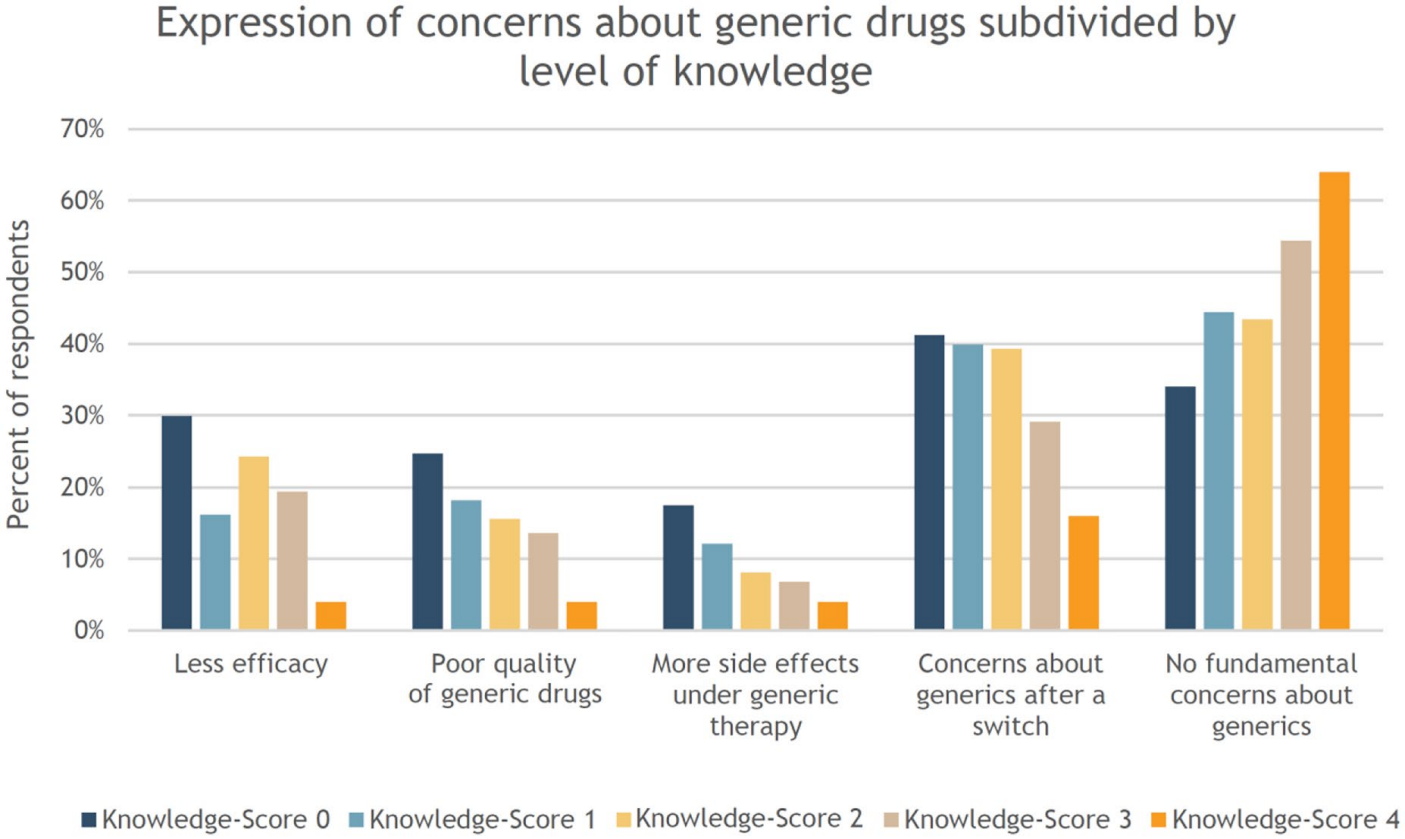
Overview of participants’ concerns about generic drugs, organized by knowledge score achieved—percentages refer to how many percent of participants with the same knowledge score selected the particular concern (for example, 30% of participants with a knowledge score of 0 selected the concern “less efficacy”)

Additionally, respondents with high knowledge scores expressed far fewer concerns. Figure 5 illustrates that participants with low knowledge are much more likely to express concerns than participants with good knowledge.

Lastly, 68.1% of all participants felt positive or highly positive about more training in the field of generics and biosimilars. 4.2% would rather not want to attend any training courses. Twenty-three percent were neutral on this subject.

## Discussion

The results of this study suggest that graduate nurses are more skeptical toward generics than hospital-employed physicians, and more often generally consider generics not to be clinically equivalent compared to the originals. Regarding biosimilars, 62.5% of all respondents were in favor of clinical equivalence to originator drugs. The attitude in this regard did not differ to a significant extent among the defined groups.

22.9% of the graduate nursing staff could not answer any question on background knowledge about generic drugs correctly. On average, the group of nursing staff achieved a knowledge score of 1.26 out of 4 possible correctly answered questions. This score was significantly lower than that of physicians from university hospitals, other hospitals, and private practices.

Regarding biosimilars, the average knowledge of the nursing staff with a score of 0.44 showed a significant drop to the knowledge of all physician groups. With regard to all three groups of physicians (university hospital, other hospitals, ordinaries), the knowledge level of graduate nurses differed significantly to the extent of medium effect size. These data suggests that, on average, graduate nurses have less knowledge regarding generic drugs and biosimilars than physicians.

The biggest concerns of the respondents related to switching from original to secondary medicines while therapy is ongoing. Only 2.8% of all physicians had concerns about generics in use as initial therapy, but 31.6% expressed doubts about switching to generics. Switching to biosimilars also caused the most worries, indicated by ¼ of the physicians. Overall, the uncertainty among nursing staff was greater than among physicians.

Only 45% of healthcare workers did not have any concerns about prescribing generics to their patients. The proportion was even lower when it comes to biosimilars, with only 34% using them without concern. However, there does not seem to be sufficient justification for the skepticism regarding biosimilars, as only around 2.5% have actually had bad experiences with biosimilars. However, the background of the bad experiences with generics needs to be investigated in the future, because negative experiences described by about 10% of the physicians and almost 15% of the nursing staff should not be neglected.

Results have also shown a clear tendency that participants with higher knowledge seem to be more convinced of the interchangeability of generics/biosimilars and have less concern than participants with lower knowledge. This suggests that most doubts are based on uncertainty and could be addressed primarily through better education on this topic.

Physicians who considered generics to be equivalent, prescribed them more often to their patients in the present study. Here, a Kendall tau-c correlation analysis was performed between the response to “1:1 interchangeability” and prescribing practices regarding generic drugs. The result showed a significant correlation of both variables to the Kendall tau-c correlation coefficient: 0.286, *p* < 0.001. A similar analysis was also performed regarding biosimilars in a smaller sample; physicians who are not used to prescribe biosimilars due to their subject-specific specialization were able to choose an alternative option to not distort the data. Indeed, the analysis regarding biosimilars also showed a significant correlation (Kendall tau-c: 0.410, *p* < 0.001). Thus, there is a significant correlation between the positive attitude toward equivalence and the willingness to prescribe secondary drugs.

A direct comparison to international study results is challenging because following comparative studies were conducted in different years, used different study designs, addressed different populations, had different questionnaires, and pursued different main objectives. Still, a general overview and indirect comparison between our study and data from Europe and the rest of the world can be made.

### Generics

A Czech study by Maly et al. specifically asked about equivalence; 60.4% of physicians acknowledged the equivalence of generic and original products [16]. In the present study from Vienna, 70.2% of physicians agreed with the equivalence.

Viennese physicians expressed fewer concerns than physicians in the review by Colgan et al., which summarized results of 52 studies from 27 countries with worldwide coverage [10]. Comparatively, the following concerns were expressed: lower quality (Vienna 14.5%, worldwide 28%), more side effects (Vienna 7.8%, worldwide 24.4%), generic switching concerns (Vienna 31.6%, worldwide 24.1%). European studies have published concerns about lower therapeutic efficacy among 14.2% of surveyed physicians in Greece [17] and 12% in Ireland [18]. Respectively, in Vienna, that figure is 16.3%. Compared to the study by Maly et al., more than twice as many Viennese physicians have had poor experiences with generic drugs in the clinic as their colleagues from the Czech Republic (9.9% versus 4.4%) [16].

Several international publications also support the finding of our study on the influence of background knowledge on personal attitudes toward generic drugs. Significant correlation analyses have also been conducted by Maly et al. and Domeyer et al. [16, 17].

### Biosimilars

14.5% of the physicians surveyed in this study did not know what biosimilars are, while 39.7% had at least heard of them or had some brief experience with them. Looking at a study from Ireland in 2017, 16% of the medical staff surveyed (¾ physicians, ¼ pharmacists) had never heard of biosimilars and another 26% could not associate anything with the term—thus 42% were not familiar with biosimilars [12]. In 2016, 59% of physicians in a survey from Malta could not relate to the term “biosimilars” or were not aware of biosimilars at all [19]. Among all physicians surveyed in a study from several countries in Latin America, 35% had never heard of or could not identify biosimilars [20]. In comparison, the results of a study among rheumatologists in Canada in 2015 were slightly more promising; only 1.2% had never heard of biosimilars, but 28.4% claimed to be unfamiliar with biosimilars [15]. Considering that, at that time, the first biosimilars gradually entered the market, the current proportions among Viennese physicians who are unaware of biosimilars are surprisingly high. An even higher figure was published in 2016 from countries in the Middle East and North Africa, where 34.2% of respondents were unaware of biosimilars [21].

Among the participants in the study from the Middle East and North Africa who were aware of biosimilars, 62% considered them to be bioequivalent to the original drug. The results among Viennese physicians are similar, with 64.7% convinced of the equivalence of biosimilars and their original reference products. However, this number is well below the average of a comparable European study from Italy in 2016, where 77% of respondents (of which ¾ were physicians and ¼ pharmacists) considered biosimilars to be equivalent in terms of efficacy and safety [22].

The correct definition of biosimilars could be identified by 50.7% of Viennese physicians. In comparison, 46% of participants could do so in a study from Russia [23], 76% from the UK [13], and visitors of the ESMO Congress 2017 (consisting of 2/3 physicians of European origin, the remaining mostly from Asia and USA) were able to choose the correct definition in 74.6% of cases [24].

A lot of international research has also been conducted on opinions regarding switching. Switching raised concerns at the following percentages: study from the USA in 2016 (55%) [14], Russia in 2019 (19%) [23], several countries in Latin America in 2015 (56%) [20], Belgium in 2017 (28% strictly against switching) [25]. In the present Vienna study, 25.7% expressed concerns about switching. In general, just 35.7% of the Viennese physicians stated that they basically had no concerns about biosimilars. This proportion is considerably higher than the number determined by a Hungarian study, where only 19.8% of the participants had no concerns [26].

In summary, the results of our survey of Viennese healthcare professionals seem to be right around the European average in terms of attitude and knowledge about biosimilars. It should additionally be noted that most of the comparative studies were published around 2016 till 2019. Since then, many biosimilars have been approved and used much more frequently in the clinic, which makes it even more critical to question the fact that there has been almost no progress in the recognition of biosimilars by healthcare professionals over time.

### Limitations

Limitations of the study include the uneven distribution of participants within the previously defined 4 study populations. For example, the population of nursing staff includes 314 participants, whereas the population of office-based physicians consists of only 57. Individual specific specialty areas of participants have been gathered, but numbers have been too low for statistical analyses and were therefore not discussed any further.

The required number of participants in the subgroups, previously determined by a sample size calculation, could not be achieved for all research questions. Regarding the Mann–Whitney *U* tests, a required sample size of 105 per group was calculated; this was not achieved in the subsequent analyses.

It was attempted to send out the questionnaire to as many hospitals and practices in Vienna as possible in order to achieve a representative study population. Nevertheless, there is a limitation in the distribution, since some medical specialties and hospitals are quantitatively more represented than others.

## Conclusion

The results of this study have shown that only just over 60% of healthcare professionals are convinced of the equivalence between generics/biosimilars and original drugs. Doubts about the efficacy, side effects, and quality persist—well under 50% of respondents stated that they had no fundamental concerns about generics or biosimilars.

In addition, the study illustrated that, even though the majority of healthcare professionals had a basic understanding of generics and can name the key differences to the original product, they generally overestimate themselves and quickly reach the limits of their knowledge when asked some more detailed questions. Even greater gaps in knowledge could be detected with regard to biosimilars—nearly 15% of all physicians did not know biosimilars at all, and almost 40% just cursorily. More than half of all participating graduate nurses had never heard of biosimilars. Overall, half of all physicians and ¾ of the nursing staff could not name the correct definition of biosimilars.

So far, internationally, nursing staff has not received much attention in this regard. However, more knowledge and education is needed here as well, because even if nurses are not involved in the prescription process, they are administering the medication to patients in hospitals, and can thus have a significant influence on patients’ views on generics and biosimilars.

Better education and training of medical staff are key to a higher and broader acceptance of generics and biosimilars. As this study has shown, the greater the knowledge, the greater the conviction of equivalence—and the greater the conviction, the greater the willingness to use generics and biosimilars in everyday clinical practice. This not only helps to reduce the costs of drugs substantially long-term, but also promotes innovative research by pharmaceutical companies into new drugs and thus continuously optimizes medical treatment options for patients.

### Supplementary Information

Below is the link to the electronic supplementary material.

Supplementary file1 (DOCX 27 KB)

Supplementary file2 (DOCX 18 KB)

Supplementary file3 (DOCX 19 KB)

Supplementary file4 (DOCX 20 KB)

## Data Availability

All data and materials that support the findings of this study are available upon request by contact with the corresponding author.
