# Supplementary material for: Perception of pharmacological equivalence of generics or biosimilars in healthcare professionals in Vienna
Source: Eur J Clin Pharmacol. 2023 Dec 22;80(3):355–66. doi: 10.1007/s00228-023-03603-3 (PMC10873459; doi:10.1007/s00228-023-03603-3)
Supplement: Supplementary file 1 — Supplementary file1 (DOCX 27 KB) [file 228_2023_3603_MOESM1_ESM.docx]

**Table S1** Questions and possible answers from the questionnaire in original order. Some questions may have been skipped due to demographic characteristics of the participants or their responses to certain questions.

| Category | Question | Answers |
| --- | --- | --- |
| Demographic data | **I am a…** | 1 = Physician 2 = Registered nurse |
| Demographic data | **Which gender do you identify with?** | 1 = Male 2 = Female 3 = Diverse 4 = No comment |
| Demographic data | **Which age group do you belong to?** | 1 = 20-29 2 = 30-39 3 = 40-49 4 = 50-59 5 = 60-69 6 = 70-79 |
| Demographic data | **Where did you complete your university degree?** | 1 = In Austria 2 = Within Europe (excluding Austria) 3 = Outside of Europe 4 = I am still studying human medicine and am therefore currently a student |
| Demographic data | **Where did you graduate as a nurse?** | 1 = In Austria 2 = Within Europe (excluding Austria) 4 = Outside of Europe 3 = I am still in training and therefore do not currently have a diploma |
| Demographic data | **In which institution do you work primarily?** | 1 = University hospital AKH Vienna 2 = Wiener Gesundheitsverbund (formerly "KAV") 5 = Viennese religious hospitals 6 = Private hospital 3 = Doctors´ office 4 = Other: ____ |
| Demographic data | **Which medical specialty do you work in?** | 1 = Internal medicine  11 = Internal Medicine → Internal Medicine and Angiology  12 = Internal Medicine → Internal Medicine and Endocrinology and Diabetology  13 = Internal Medicine → Internal Medicine and Gastroenterology  14 = Internal Medicine → Internal Medicine and Hematology and Oncology  15 = Internal medicine → Internal medicine and Cardiology  16 = Internal medicine → Internal medicine and Nephrology  17 = Internal medicine → Internal medicine and Pneumology  18 = Internal medicine → Internal medicine and Rheumatology  19 = Internal Medicine → Internal Medicine and Infectiology  110 = Internal medicine → Internal medicine and Intensive Care Medicine  2 = Other specialties  21 = Other specialties → General medicine  22 = Other specialties → Ophthalmology  23 = Other specialties → Gynaecology and obstetrics  24 = Other specialties → Otorhinolaryngology  25 = Other specialties → Skin and sexually transmitted diseases  26 = Other specialties → Pediatrics and adolescent medicine  27 = Other specialties → Neurology  28 = Other specialties → Pathology  29 = Other specialties → Clinical pharmacology  210 = Other specialties → Psychiatry  211 = Other specialties → Radiology  212 = Other specialties → Radiation oncology  213 = Other specialties → Urology  214 = Other specialties → Anesthesiology and Intensive Care Medicine  215 = Other specialties → Physical medicine and general rehabilitation  216 = Other specialties → Transfusion medicine  3 = Surgery  31 = Surgery → General surgery  32 = Surgery → Cardiovascular surgery  33 = Surgery → Pediatric surgery  34 = Surgery → Orthopaedics and Trauma Surgery  35 = Surgery → Plastic and Aesthetic Surgery  36 = Surgery → Thoracic surgery  37 = Surgery → Visceral surgery  38 = Surgery → Neurosurgery  39 = Surgery → Oral and maxillofacial surgery  4 = Other: ____ |
| Demographic data | **What is your current level of qualification?** | 1 = Basic training  2 = General practitioner training  3 = Specialist training  7 = General practitioner  4 = Specialist doctor  5 = Senior physician  6 = Head of department |
| Perception towards generics | **Are you convinced of the equivalence of generics compared to the original drugs?** | 1 = Yes 2 = No |
| Perception towards generics | **How much do you agree with the following statement "generics and original product are 1:1 interchangeable"?** | Scale: 1-11 (1 = not agree at all 11 = fully agree) |
| Perception towards generics | **Do you prescribe generics?** | 1 = Yes, as often as possible. I usually choose a generic when starting a new treatment. I also like to switch to a generic if the patient is already taking an original drug  2 = I usually prescribe a generic for a new therapy, but I normally leave the original for an existing therapy  3 = I am cautious in this regard. It depends on the indication, specific therapy, and the patient's condition. I only prescribe a generic if all the variables are met for me  4 = I always try to prescribe the original, even for new prescriptions; I tend to avoid generics  5 = Never |
| Perception towards generics | **Do you have concerns about generics compared to the original medicines?** | 1 = I have concerns about more side effects with generic therapy  2 = I have concerns about reduced efficacy  3 = I worry about the poor quality of generics  4 = I have had bad experiences with the use of generics  5 = I have concerns about the use of generics as first-line therapy  6 = I have concerns about the use of generics in a switch (switching to a generic during ongoing therapy)  7 = I generally have no concerns about the clinical use of generics  8 = Other concerns: ____  9 = Don't know |
| Perception towards generics | **Would you currently prefer to use the original product for self-treatment rather than a generic?** | 1 = Yes, I would prefer the original drug  2 = No, I would not prefer the original drug  3 = No comment |
| Perception towards generics | **How would you rate your level of knowledge about generics on a scale of 1-10?** | Scale: 1-10  (1 = I have no knowledge at all  10 = I have a lot of knowledge ) |
| Knowledge about generics | **Are generic products permitted to differ from the original product?** | 1 = There must be no differences in the composition of the two drugs  2 = (Correct) The active ingredient must be the same, the galenics and excipients may differ  3 = The active ingredient may differ, but all excipients added as well as the manufacturing process must be the same  4 = There are no strict guidelines on similar ingredients, but the same effect must be proven by studies  5 = Don´t know |
| Knowledge about generics | **How is the similar or equivalent efficacy verified?** | 1 = (Correct) The concentration curve of the active substance in the blood is examined, the curve must be within a defined range in order to conclude clinical equivalence  2 = Equivalence is determined by testing on volunteers and later on patients. The clinical effect and side effects must not differ  3 = In the laboratory, the binding to the specific receptor is biochemically tested. Changes in the pathway and receptor activities must occur to the same extent for the original and the generic drug  4 = It is checked whether the chemical composition is exactly the same as in the respective original preparation. Once this is assured, no further tests are necessary  5 = Don´t know |
| Knowledge about generics | **The following applies to generic drugs:** | 1 = They have been proven to be less safe than the original drug  2 = Only one generic drug at a time may be produced by a single company for a drug whose patent has expired  3 = They may be developed and marketed after the expiry of the legally determined patent period of 6 months after the market entry of the original medicinal product  4 = (Correct) Generic drugs are subject to a uniformly defined quality testing procedure. However, stricter guidelines than usual apply to generics with a narrow therapeutic range  5 = Don´t know |
| Knowledge about generics | **How high is the price of generics compared to the original product?** | 1 = The newer generics are often more expensive than the original drug  2 = The price of generic drugs is comparable to the original price  3 = The price of generic drugs is always cheaper than the original. The discount depends on supply and demand  4 = (Correct) A tier plan specifies exactly how much cheaper the generic must be compared to the original  5 = Don´t know |
| Perception towards biosimilars | **Are you familiar with the term “biosimilars”?** | 1 = Yes  2 = I have heard of them or have had brief contact with them through training/profession  3 = No, I don't know this group or can't imagine what the term means |
| Perception towards biosimilars | **Are you convinced of the equivalence of biosimilars compared to the original drugs?** | 1 = Yes 2 = No |
| Perception towards biosimilars | **How much do you agree with the following statement "biosimilars and original product are 1:1 interchangeable"?** | Scale: 1-11 (1 = not agree at all 11 = fully agree) |
| Perception towards biosimilars | **Do you prescribe biosimilars?** | 1 = Yes, as often as possible. I usually choose a biosimilar when starting a new treatment. I also like to switch to a biosimilar for an existing therapy with an original drug  2 = I usually prescribe a biosimilar for a new therapy, but I usually leave the original for an existing therapy  3 = I am cautious in this regard. It depends on the indication, specific therapy, and condition of the patient. I only prescribe a biosimilar if all the variables are met for me  4 = I always try to prescribe the original, even for new prescriptions; I tend to avoid biosimilars  5 = Never  6 = I hardly ever deal with biosimilars in my area of specialization and therefore cannot make a statement on this subject |
| Perception towards biosimilars | **Do you have concerns about biosimilars compared to the original medicines?** | 1 = I have concerns about more side effects with biosimilars in therapy  2 = I have concerns about reduced efficacy  3 = I worry about the poor quality of biosimilars  4 = I have had bad experiences with the use of biosimilars  5 = I have concerns about the use of biosimilars as first-line therapy  6 = I have concerns about the use of biosimilars in a switch (switching to a biosimilar during ongoing therapy)  7 = I generally have no concerns about the clinical use of biosimilar  8 = Other concerns: ____  9 = Don't know |
| Perception towards biosimilars | **Would you currently prefer to use the original product for self-treatment rather than a biosimilar?** | 1 = Yes, I would prefer the original drug  2 = No, I would not prefer the original drug  3 = No comment |
| Perception towards biosimilars | **How would you rate your level of knowledge about biosimilars on a scale of 1-10?** | Scale: 1-10  (1 = I have no knowledge at all  10 = I have a lot of knowledge ) |
| Knowledge about biosimilars | **The following applies to biosimilars:** | 1 = Biosimilars are purely herbal preparations from the field of homeopathy  2 = The only requirement for approval of a biosimilar on the European market is a bioequivalence study  3 = (Correct) Biosimilars are derivative products of biopharmaceuticals, however not with structurally identical, but highly similar active ingredient  4 = All production batches are identical with the same manufacturing process every time  5 = Don´t know |
| Knowledge about biosimilars | **How high are the development expenses for biosimilars compared to those for generics?**  **The costs for biosimilars are...** | 1 = (Correct) Higher  2 = About the same  3 = Lower  4 = Don´t know |
| Knowledge about biosimilars | **How are biosimilars different from generics?** | 1 = Biosimilars are significantly older drugs and have not been sufficiently proven in terms of safety and efficacy; they are now gradually being replaced by generics in many specialties  2 = There are more biosimilars on the European market than generics  3 = (Correct) Generics consist of comparatively small molecules, while biosimilars are large, complex proteins  4 = Biosimilars, unlike generics, are manufactured from purely biological resources  5 = Don´t know |
| Knowledge about biosimilars | **How are biosimilars manufactured?** | 1 = Biosimilars are produced chemically in the laboratory. Using large-scale cell cultures, it is possible to obtain modified T-cell lines that can then be used therapeutically in biosimilars.  2 = Biosimilars are naturally occurring substances that can be extracted from plants and mushrooms and are then processed into homeopathic substances  3 = The production of biosimilars is subject to a biological fermentation process. Through different fermentation procedures, in combination with specific RNA sequences, biosimilars are synthesized chemically in the laboratory  4 = (Correct) Biosimilars are produced by a biological organism; the organism incorporates inserted DNA into its genome and subsequently produces proteins that can then be harvested  5 = Don´t know |
| Education | **Would you be interested in more training on the topic of generics and biosimilars?** | 1 = :(( 2 = :( 3 = :\| 4 = :) 5 = :)) |
