# Supplementary material for: Perception of pharmacological equivalence of generics or biosimilars in healthcare professionals in Vienna
Source: Eur J Clin Pharmacol. 2023 Dec 22;80(3):355–66. doi: 10.1007/s00228-023-03603-3 (PMC10873459; doi:10.1007/s00228-023-03603-3)
Supplement: Supplementary file 2 — Supplementary file2 (DOCX 18 KB) [file 228_2023_3603_MOESM2_ESM.docx]

**Table S2** Demographic data of participants.

The most frequently represented specialties within the "other specialties", which includes both doctors and nursing staff, are Anesthesia and intensive care (50), psychiatry (45), pediatrics and adolescent medicine (39), ear, nose and throat medicine (28), neurology (25) and gynecology (18).

|  |  | Physicians (n=282) | Nurses (n=314) |
| --- | --- | --- | --- |
| **Sex** | Male | 155 (55.0%) | 52 (16.6%) |
|  | Female | 126 (44.6%) | 261 (83.1%) |
|  | Not specified | 1 (0.4%) | 1 (0.3%) |
| **Age** | 20-29 | 27 (9.6%) | 40 (12.7%) |
|  | 30-39 | 51 (18.1%) | 65 (20.7%) |
|  | 40-49 | 46 (16.3%) | 80 (25.5%) |
|  | 50-59 | 81 (28.7%) | 111 (35.4%) |
|  | 60-69 | 56 (20.0%) | 14 (4.5%) |
|  | 70-79 | 14 (5.0%) | 0 (0%) |
|  | Not specified | 7 (2.5%) | 4 (1.3%) |
| **Location of medical education** | Austria | 266 (94.3%) | 292 (93.0%) |
|  | Within Europe | 14 (5.0%) | 21 (6.7 %) |
|  | Outside Europe | 2 (0.7%) | 1 (0.3%) |
| **Primary working area** | University hospital Vienna | 67 (23.8%) | 110 (35.0%) |
|  | Public hospital association “Wiener Gesundheitsverbund“ | 113 (40.1%) | 193 (61.5%) |
|  | Doctors´ offices in Vienna | 57 (20.2%) | 0 (0%) |
|  | Vienna religious hospital associations | 28 (10%) | 7 (2.2%) |
|  | Vienna private hospitals | 7 (2.5%) | 3 (1%) |
|  | Other healthcare facilities | 10 (3.5%) | 1 (0.3%) |
| **Medical field** | Internal Medicine | 74 (26.2%) | 95 (30.3%) |
|  | Surgery | 21 (7.4%) | 69 (22.0%) |
|  | General Medicine | 42 (14.9%) | 2 (0.6%) |
|  | Other Specialties | 128 (45.4%) | 129 (41.1%) |
|  | Not specified | 17 (6.0%) | 19 (6.1%) |
| **Education degree** | Specialist-in-training | 54 (19.1%) |  |
|  | Certified Specialist/General Practitioner | 105 (37.2%) |  |
|  | Senior Physician | 100 (35.5%) |  |
|  | Chief Physician | 23 (8.2%) |  |
