# Supplementary material for: Perception of pharmacological equivalence of generics or biosimilars in healthcare professionals in Vienna
Source: Eur J Clin Pharmacol. 2023 Dec 22;80(3):355–66. doi: 10.1007/s00228-023-03603-3 (PMC10873459; doi:10.1007/s00228-023-03603-3)
Supplement: Supplementary file 3 — Supplementary file3 (DOCX 19 KB) [file 228_2023_3603_MOESM3_ESM.docx]

**Table S3** Overview of the knowledge check on generic drugs; the correct answer for each case is marked accordingly. The percentages refer to the total number of the respective group - physicians or nurses.

| Question | Answer Options | Physicians | Nurses |
| --- | --- | --- | --- |
| Are generic products permitted to differ from the original product? | - There must be no differences in the composition of the two drugs | n=48 (17.0%) | n=78 (24.8%) |
|  | - ***(Correct) The active ingredient must be the same, the galenics and excipients may differ*** | ***n=215 (76.2%)*** | ***n=200 (63.7%)*** |
|  | - The active ingredient may differ, but all excipients added as well as the manufacturing process must be the same | n=2 (0.7%) | n=3 (1.0%) |
|  | - There are no strict guidelines on similar ingredients, but the same effect must be proven by studies | n=11 (3.9%) | n=16 (5.1%) |
|  | - Don´t know | n=6 (2.1%) | n=17 (5.4%) |
| How is the similar or equivalent efficacy verified? | - ***(Correct) The concentration curve of the active substance in the blood is examined, the curve must be within a defined range in order to conclude clinical equivalence*** | ***n=110 (39.0%)*** | ***n=42 (13.4%)*** |
|  | - Equivalence is determined by testing on volunteers and later on patients. The clinical effect and side effects must not differ | n=42 (14.9%) | n=47 (15.0%) |
|  | - In the laboratory, the binding to the specific receptor is biochemically tested. Changes in the pathway and receptor activities must occur to the same extent for the original and the generic drug. | n=30 (10.6%) | n=54 (17.2%) |
|  | - It is checked whether the chemical composition is exactly the same as in the respective original preparation. Once this is assured, no further tests are necessary | n=35 (12.4%) | n=28 (8.9%) |
|  | - Don´t know | n=65 (23.0%) | n=143 (45.5%) |
| The following applies to generic drugs: | - They have been proven to be less safe than the original drug | n=3 (1.1%) | n=4 (1.3%) |
|  | - Only one generic drug at a time may be produced by a single company for a drug whose patent has expired | n=6 (2.1%) | n=12 (3.8%) |
|  | - They may be developed and marketed after the expiry of the legally determined patent period of 6 months after the market entry of the original medicinal product | n=72 (25.5%) | n=101 (32.2%) |
|  | - ***(Correct) Generic drugs are subject to a uniformly defined quality testing procedure. However, stricter guidelines than usual apply to generics with a narrow therapeutic range*** | ***n=147 (52.1%)*** | ***n=93 (29.6%)*** |
|  | - Don´t know | n=54 (19.1%) | n=104 (33.1%) |
| How high is the price of generics compared to the original product? | - The newer generics are often more expensive than the original drug | n=2 (0.7%) | n=3 (1.0%) |
|  | - The price of generic drugs is comparable to the original price | n=9 (3.2%) | n=18 (5.7%) |
|  | - The price of generic drugs is always cheaper than the original. The discount depends on supply and demand | n=157 (55.7%) | n=166 (52.9%) |
|  | - ***(Correct) A tier plan specifies exactly how much cheaper the generic must be compared to the original*** | ***n=84 (29.8%)*** | ***n=62 (19.7%)*** |
|  | - Don´t know | n=30 (10.6%) | n=65 (20.7%) |
