# Supplementary material for: Perception of pharmacological equivalence of generics or biosimilars in healthcare professionals in Vienna
Source: Eur J Clin Pharmacol. 2023 Dec 22;80(3):355–66. doi: 10.1007/s00228-023-03603-3 (PMC10873459; doi:10.1007/s00228-023-03603-3)
Supplement: Supplementary file 4 — Supplementary file4 (DOCX 20 KB) [file 228_2023_3603_MOESM4_ESM.docx]

**Table S4** Overview of the knowledge check on biosimilar drugs; the correct answer for each case is marked accordingly. The percentages refer to the total number of the respective group - physicians or nurses.

| Question | Answer Options | Physicians | Nurses |
| --- | --- | --- | --- |
| The following applies to biosimilars: | - Biosimilars are purely herbal preparations from the field of homeopathy | n=0 (0.0%) | n=0 (0.0%) |
|  | - The only requirement for approval of a biosimilar on the European market is a bioequivalence study | n=20 (7.1%) | n=2 (0.6%) |
|  | - ***(Correct) Biosimilars are derivative products of biopharmaceuticals, however not with structurally identical, but highly similar active ingredient*** | ***n=143 (50.7%)*** | ***n=81 (25.8%)*** |
|  | - All production batches are identical with the same manufacturing process every time | n=10 (3.5%) | n=2 (0.6%) |
|  | - Don´t know | n=68 (24.1%) | n=50 (15.9%) |
|  | - Biosimilars unknown | n=41 (14.5%) | n=179 (57.0%) |
| How high are the development expenses for biosimilars compared to those for generics? | - ***(Correct) Higher*** | ***n=54 (19.1%)*** | ***n=18 (5.7%)*** |
| The costs for biosimilars are... | - About the same | n=21 (7.4%) | n=12 (3.8%) |
|  | - Lower | n=82 (29.1%) | n=38 (12.1%) |
|  | - Don´t know | n=84 (29.8%) | n=67 (21.3%) |
|  | - Biosimilars unknown | n=41 (14.5%) | n=179 (57.0%) |
| How are biosimilars different from generics? | - Biosimilars are significantly older drugs and have not been sufficiently proven in terms of safety and efficacy; they are now gradually being replaced by generics in many specialties | n=2 (0.7%) | n=0 (0.0%) |
|  | - There are more biosimilars on the European market than generics | n=11 (3.9%) | n=1 (0.3%) |
|  | - ***(Correct) Generics consist of comparatively small molecules, while biosimilars are large, complex proteins*** | ***n=91 (32.3%)*** | ***n=16 (5.1%)*** |
|  | - Biosimilars, unlike generics, are manufactured from purely biological resources | n=30 (10.6%) | n=36 (11.5%) |
|  | - Don´t know | n=107 (37.9%) | n=82 (26.1%) |
|  | - Biosimilars unkown | n=41 (14.5%) | n=179 (57.0%) |
| How are biosimilars manufactured? | - Biosimilars are produced chemically in the laboratory. Using large-scale cell cultures, it is possible to obtain modified T-cell lines that can then be used therapeutically in biosimilars. | n=11 (3.9%) | n=8 (2.5%) |
|  | - Biosimilars are naturally occurring substances that can be extracted from plants and mushrooms and are then processed into homeopathic substances | n=5 (1.8%) | n=4 (1.3%) |
|  | - The production of biosimilars is subject to a biological fermentation process. Through different fermentation procedures, in combination with specific RNA sequences, biosimilars are synthesized chemically in the laboratory | n=6 (2.1%) | n=4 (1.3%) |
|  | - ***(Correct) Biosimilars are produced by a biological organism; the organism incorporates inserted DNA into its genome and subsequently produces proteins that can then be harvested*** | ***n=91 (32.3%)*** | ***n=24 (7.6%)*** |
|  | - Don´t know | n=128 (45.4%) | n=95 (30.3%) |
|  | - Biosimilars unknown | n=41 (14.5%) | n=179 (57.0%) |
